# Quantitative multimodal imaging of extensive macular atrophy with pseudodrusen and geographic atrophy with diffuse trickling pattern

**DOI:** 10.1038/s41598-023-28906-4

**Published:** 2023-02-01

**Authors:** Alessio Antropoli, Alessandro Arrigo, Lorenzo Bianco, Alessandro Berni, La Franca Lamberto, Andrea Saladino, Francesco Bandello, Maurizio Battaglia Parodi

**Affiliations:** IRCCS San Raffaele Scientific Institute, Vita-Salute San Raffaele University, Via Olgettina 60, 20132 Milan, Italy

**Keywords:** Diagnostic markers, Eye diseases, Macular degeneration

## Abstract

To compare clinical and imaging characteristics of extensive macular atrophy with pseudodrusen-like appearance (EMAP) versus diffuse-trickling geographic atrophy (DTGA) and non-diffuse-trickling geographic atrophy (nDTGA) phenotypes of age-related macular degeneration. Prospective, observational study performed in the Ophthalmology Department of IRCCS San Raffaele Hospital between January 2015 and January 2021. Patients examination included fundus autofluorescence (FAF) and optical coherence tomography at baseline and follow-up visits. We measured subfoveal choroidal thickness (SCT), Sattler/choroid ratio (SCR), choroidal vascularity index and ellipsoid zone disruption distance on OCT scans. We calculated progression rates and circularity of the atrophic lesions on FAF images. These variables were compared between the three groups and correlations with progression rates and visual acuity were assessed. Sixty-three eyes from 63 patients were included: 18 with EMAP, 18 with DTGA and 27 with nDTGA. Mean follow-up was 3.73 ± 2.12 years. EMAP and DTGA shared a faster progression, lower circularity and SCR, and higher EZ disruption distance than nDTGA, while SCT and CVI were similar between the three groups. Baseline circularity and SCR correlated with progression rates. EMAP and DTGA show similar OCT and FAF characteristics, which differ from nDTGA.

## Introduction

Extensive macular atrophy with pseudodrusen-like appearance (EMAP) is a rare cause of bilateral symmetric macular atrophy, affecting patients at the beginning of their sixth decade. EMAP is characterized by a polycyclic morphology with a major vertical axis, pseudodrusen up to mid-periphery, and paving stone degeneration in the far periphery^1^. Night blindness is a frequent sign at presentation and the progression is fast, with early foveal involvement. Results from a case–control study with 115 EMAP patients suggest that a family history of age-related macular degeneration (AMD), glaucoma, female sex, and several biomarkers of systemic inflammation and abnormal complement pathway regulation are associated with an increased risk of developing EMAP, while AMD-associated cardiovascular risk factors are not^2^. The same research group recently highlighted a possible toxic etiology, leading to pseudodrusen formation and cone apoptosis^2^. Despite these distinctive characteristics, it may be difficult to distinguish macular atrophy secondary to EMAP from the diffuse-trickling geographic atrophy (DTGA) pattern, which is considered the most aggressive form of GA secondary to AMD, since both conditions share many overlapping features: a relatively young age at presentation, bilaterality, fast progression, lobular and grayish aspect of the atrophic area on fundus autofluorescence (FAF), and predominance of pseudodrusen^3,4^. Furthermore, a diffuse splitting of the Bruch-RPE complex has been reported in both EMAP and DTGA^3,5^.

In the past few years, the alternative complement pathway has been implicated in the pathogenesis of AMD and GA^6–8^. Many recent clinical trials have evaluated the efficacy of complement inhibitors on slowing the progression of AMD^9^. However, ongoing clinical trials only enroll individuals with a clinical diagnosis of AMD and do not distinguish between various macular atrophy subtypes, potentially excluding individuals with conditions that would benefit from such interventions (i.e. EMAP). Recently, the the choroid has also been implicated in GA progression^10,11^, but its modifications in different macular atrophy patterns, as well as in EMAP, have not been thoroughly examined.

This led us to design a prospective study to compare clinical and imaging characteristics of macular atrophy in EMAP versus GA phenotypes of AMD.

## Methods

We planned a prospective study in January 2015 recruiting patients affected by macular atrophy who were progressively registered in our database till January 2018. All the patients affected by EMAP, DTGA or non-diffuse-trickling geographic atrophy (nDTGA) were enrolled in the study, with a planned examination visit every 12 months. All the recruited patients underwent an ophthalmological examination complete of BCVA measurement, using Early Treatment Diabetic Retinopathy Study (ETDRS) charts; slit-lamp biomicroscopy with fundus examination; IOP measurement with Goldmann tonometer; and FAF and optical coherence tomography (OCT) imaging, using a Spectralis HRA + OCT (Heidelberg Engineering, Germany). We also investigated the presence of cardiovascular (CV) risk factors (including hypertension, hypercholesterolaemia, stroke, heart failure, heart attack, and cardiac rhythm diseases) and a history of glaucoma.

The research followed the tenets of the Declaration of Helsinki and was approved by the ethical committee of IRCCS Scientific Institute San Raffaele Hospital. Informed consent was obtained from all recruited subjects.

### Study participants

The study included patients with macular atrophy secondary to EMAP, DTGA, or nDTGA. Diagnosis of EMAP was defined when all the following criteria were met: (1) presence of macular atrophy with symmetric, polycyclic borders, major vertical axis; (2) pseudodrusen-like deposits surrounding the atrophic lesion; (3) peripheral paving-stone degeneration; (4) age under 55 years at the onset of visual symptoms (either nyctalopia, reduced vision or methamorphopsias).

Patients diagnosed with macular atrophy due to AMD were sub-classified on the basis of their appearance on FAF imaging as described by Holz et al.^4^ In brief, patients were assigned to the DTGA group if the atrophic area met all of the following criteria: (1) age of at least 55 years at symptom onset; (2) diffuse increased AF signal extending beyond the edges of the atrophy; (3) coalescent, greyish, lobular aspect.

**Figure 1:**
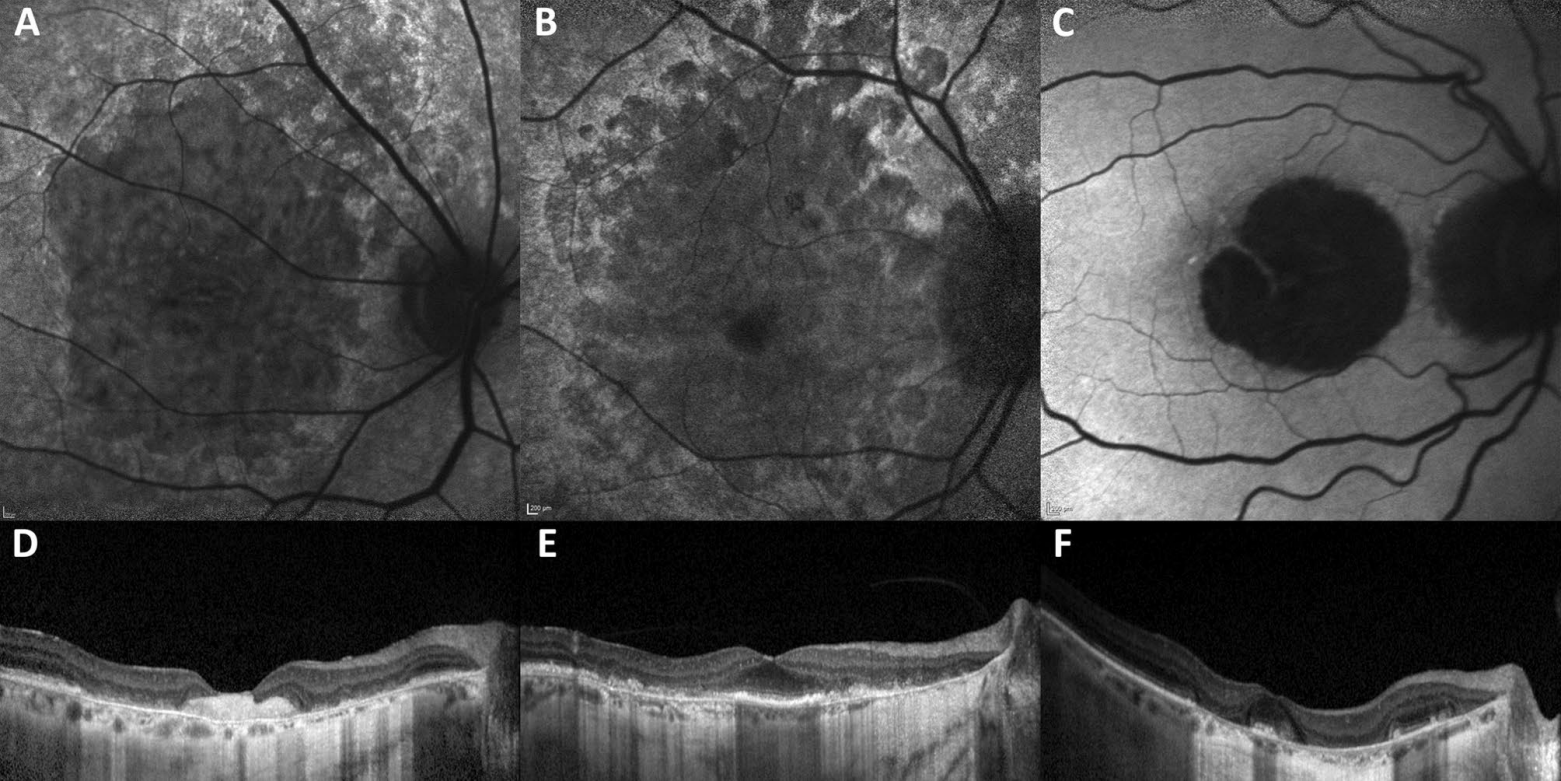
Multimodal imaging of each condition. Blue-light fundus autofluorescence and optical coherence tomography of (**A**, **D**) Extensive macular atrophy with pseudodrusen-like appearance, (**B**, **E**) diffuse-trickling geographic atrophy and (**C**, **F**) non-diffuse-trickling geographic atrophy.

AMD patients with macular atrophy aspect not matching criteria (2) or (3) were assigned to the nDTGA group (Fig. 1).

A senior grader (M.B.P.) confirmed the diagnosis of cases that met the requirements of one of the three groups.

Exclusion criteria were: minimum follow-up of less than 12 months, high media opacity, fixation inadequate for high-quality imaging, systemic or other ocular conditions known to cause chorioretinal atrophy, history of neovascular complications, or administration of intravitreal anti-VEGF agents. When both eyes of the same patients were eligible for inclusion in the study, only a single eye was randomly selected.

### Acquisition protocol, imaging analysis and outcome variables

Structural OCT images were acquired using a Spectralis HRA + OCT (Heidelberg Engineering, Heidelberg, Germany). The standard acquisition protocol comprised at least a radial pattern of six B-scans with a 30° angle and a high number of frames (ART > 25) and a raster pattern of nineteen 20° angle B-scans (ART > 9) with a spacing of 234 µm. The enhanced depth imaging (EDI) modality was selected to provide a better visualization of the choroid.

The area of atrophy at baseline and at the last available follow-up visit was measured on FAF images with blue light (30° × 30° field of view) centered on the fovea using the semi-automated region finder tool provided by Heidelberg HEYEX software^12,13^. OCT scans and infrared images supported the identification and confirmed the presence of foveal involvement and extension of atrophic areas, as recommended by classification of atrophy reports^14,15^. The following parameters were provided by the software: total atrophic area, change from baseline examination, rate of change in mm^2^ per year. A quantitative analysis of the circularity index was then performed using FAF images and corresponding masks of the atrophic areas, exported as .bmp files from the region finder and imported to ImageJ software (National Institute of Health, Bethesda, MD). The image scale was set by using HEYEX software to match dimensions in millimeters and pixels for every FAF image. The contour of the atrophic area exported by the region finder was automatically selected using the tracing tool (instead of manually drawing it) on the mask image and saved as a “region of interest” (ROI). The ROI was then superimposed on the original FAF image and the area, perimeter and circularity were calculated using the “measure” command (this method had displayed excellent inter-rater reliability in a previous study)^16^ We thus obtained the following variables for every eye included in the study, both at baseline and at the final follow-up examination:

- Area of atrophy (mm^2^)
- Progression rate (mm^2^/y), calculated by subtracting the area at baseline from the area at follow-up and dividing by the overall length of observation in years^17^.
- Progression rate after square-root transformation of the area measurements (mm/y), to reduce the dependence on the baseline size of the atrophic lesion^18^.
- Circularity, whose formula is 4π*area/ perimeter^2^, identifies irregular shapes with values closer to 0^19^.

### Choroidal variables and outcomes

Subfoveal and mean choroidal thickness (CT), Haller layer thickness (HLT) and Sattler layer-choriocapillaris complex thicknesses (SLCCT), and choroidal vascularity index (CVI) at baseline and at the end of the follow-up were measured on OCT B-scans centered on the fovea, in accordance with the current literature^11,20,21^. Large choroidal vessels defined the Haller layer, whereas SLCCT was defined as the distance between the lower border of the retinal pigment epithelium–Bruch complex and the upper border of the Haller layer, as previously reported^11^.

All choroidal measurements were taken by two trained graders (LB and AS). In particular, subfoveal CT was manually measured between the Bruch membrane and the sclerochoroidal interface in the subfoveal location, and at 0.5-mm intervals, from 1.0 mm nasal to 1.0 mm temporal to the fovea; whereas mean CT, HLT and SLCCT were calculated from the mean value of five samples (subfoveal, 750 mm [right- left] and 1500 mm [right-left]).

Given that patients with EMAP, DTGA and nDTGA belong by definition to different age groups, and that choroidal thickness reduces with age^22^. we also calculated the ratio of the SLCC on mean CT (i.e. Sattler/Choroid ratio [SCR]) as an additional parameter, so as to standardize our results.

**Figure 2:**
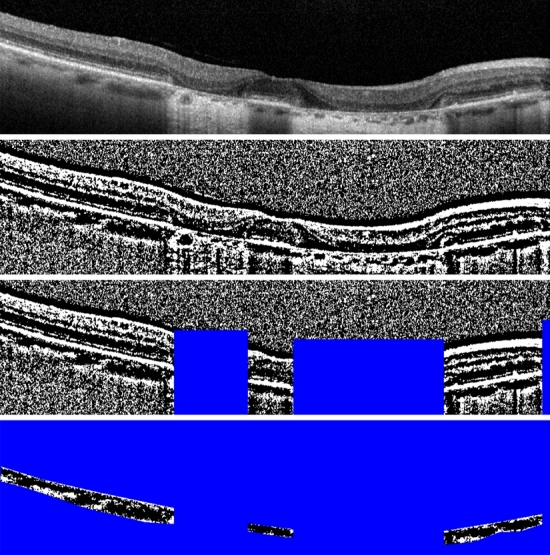
Image processing for calculation of choroidal vascularity index (CVI). OCT B-scans were imported into ImageJ software (National Institute of Health, Bethesda, MD) and binarized with Niblack’s autolocal threshold. Areas of hyper- and hypo-transmission were excluded from the final ROI. In this case CVI is 0.69.

CVI was calculated as already described in the literature^21^. Specifically, after importing the scans into the ImageJ software, we calculated the CVI by selecting the whole choroid as ROI, since the CVI is not affected by the region considered^23^. The Bruch membrane and the sclerochoroidal junction represented the upper and lower boundaries of the region. Areas of hyper- and hypo-transmission were also included in the ROI. The entire image was then converted into 8-bit form, binarized with Niblack’s autolocal threshold and then reconverted into RGB to isolate the choroidal area. The ROIs including the hyper- and hypo-transmission areas were selected and deleted from the choroidal area to be analyzed, in order to avoid under- and over-estimating the CVI (see Fig. 2). After the binarization, the CVI was obtained through an in-house plug-in that automatically calculated the ratio of the luminal choroidal area (black pixels) to the total choroidal area (black pixels + white pixels).

**Figure 3:**
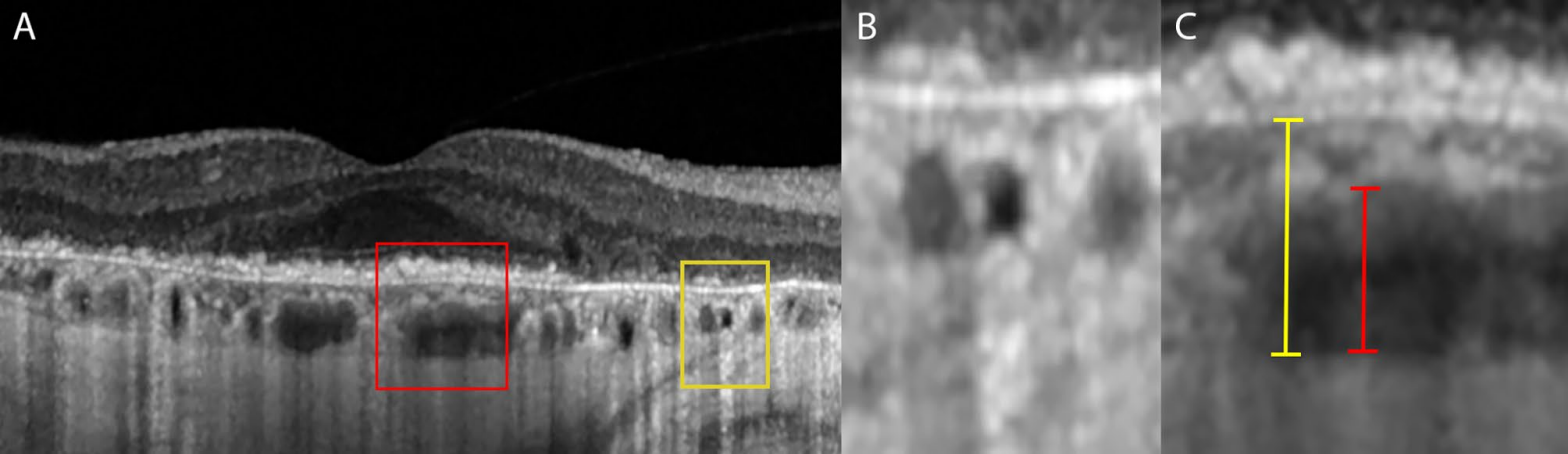
(**A**) Choroidal cavern visible on structural OCT, together with examples of choroidal thickness measurements. (**B**) Detail of the yellow box: choroidal cavern visible as a non-reflective, round structure with a typical hypertransmission tail. (**C**) Detail of the red box: Sattler layer-choriocapillaris complex thicknesses results from the difference between choroidal thickness (yellow marker) and Haller layer thickness (red marker).

Lastly, IR + OCT scans were reviewed for the presence of choroidal lipid globule caverns, which are defined as non-reflective spherical to polyhedral structures with a posterior hypertransmission tail on structural OCT, often hyperreflective on IR images in cases of RPE loss, and have been detected in association with RPE atrophy in AMD (Fig. 3)^24–26^. These caverns were first identified as hyperreflective dots in IR images^24^, and subsequently were confirmed on the corresponding OCT scans.

### Retinal variables and outcomes

**Figure 4:**
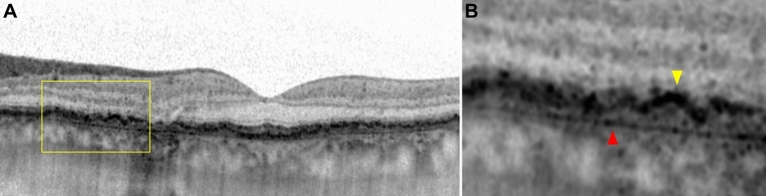
OCT B-Scan of a DTGA patient, showing: (**A**) a diffuse splitting of the Bruch-RPE complex, and (**B**) close-up of yellow box, with Bruch’s membrane (red arrow) and RPE (yellow arrow).

**Figure 5:**
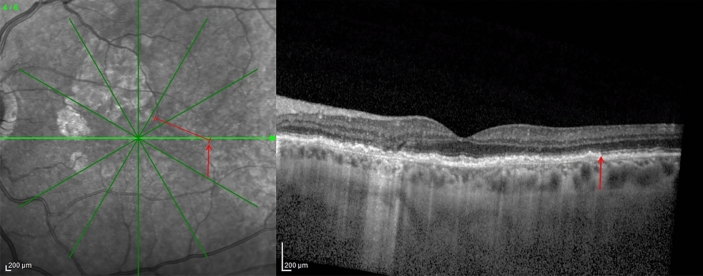
IR + OCT image showing a diffuse Bruch-RPE splitting. After identifying on the OCT B-scan the point where the ellipsoid zone faded, its distance from the closest edge of the atrophy was measured on the corresponding IR image (1856 μm in this case).

A diffuse Bruch-RPE splitting has been described in both EMAP and DTGA^3,5^, therefore we designed a surrogate quantitative parameter for this phenomenon, and called it “EZ disruption distance”. On horizontal OCT scans, we checked the presence of a Bruch-RPE splitting at the edges of the atrophic lesion (Fig. 4). Then, on the temporal side of horizontal IR + OCT 30° scans of patients displaying this feature, we identified the point where the ellipsoid zone (EZ) faded where the Bruch-RPE complex split. On the corresponding IR image, we measured the distance between that point and the closest edge of the atrophic lesion (Fig. 5). The temporal side was chosen because of the presence of the optic disc on the nasal side and the frequent extension of the atrophy beyond 30° along the vertical axis. All measurements were taken by two graders (L.B. and A.S.) and intraclass correlation coefficient was calculated to assess intergrader agreement.

When Bruch-RPE splitting at baseline was absent or extended beyond the total length of the horizontal OCT scan, it was not considered possible to gauge the EZ disruption distance in that eye, whereas if this feature disappeared over the course of the follow-up, the disruption distance was held to have progressed to 0 µm.

### Statistical analysis

All descriptive data were expressed as mean ± standard deviation (SD) for continuous normally distributed variables, as median (interquartile range, IQR) for non-normal ones, and as frequency and percentages for categorical ones. Testing for normality of continuous variables was assessed using the Kolmogorov–Smirnov test. Comparison of frequencies among groups was performed using the chi-square test. Differences between baseline and follow-up measurements of continuous variables were assessed using the paired samples t-test. We adopted Pearson’s correlation test for circularity and Spearman’s correlation test for baseline SCR and CVI to assess the relationship with rates of atrophy progression and BCVA loss.

For the purposes of statistical analysis, we merged all GA patterns except DTGA, meaning we compared 3 subgroups: EMAP, DTGA and non-diffuse-trickling geographic atrophy (nDTGA). Continuous normally distributed variables were compared between the EMAP and the other groups using the Tukey HSD post hoc test for ANOVA; non-normal variables were compared using the Kruskal–Wallis test.

All tests were two-sided and the level of significance was set at *p* < 0.05. Analyses were performed using SPSS Statistics 23 (IBM; Armonk, NY).

## Results

Overall, 560 patients affected by macular atrophy were considered for the study. Three hundred and sixty-two patients were excluded because affected by other subforms of macular atrophy. One hundred and thirty-five patients were ruled out either because they refused to take part in our study (87 patients), or because of poor quality imaging (48 patients). A total of 63 eyes from 63 patients were included in the analyses (18 with EMAP, 18 with DTGA, 27 with nDTGA).

Fifty-one patients had a regular yearly follow-up examination (± 1 month), whereas 12 patients had an uneven follow-up, ranging from 14 to 24 months, with an average visit every 16.8 months, especially because of the COVID-19 restrictions.

Thirty patients were female (47.6%): 4 (22.2%) in the EMAP group, 14 (77.8%) in the DTGA group and 12 (44.4%) in the nDTGA group. Age at baseline differed significantly between the 3 groups (all *p* < 0.001): EMAP patients were the youngest group (55.4 ± 2.5 years), followed by DTGA (65.4 ± 6.1 years) and lastly nDTGA patients (77.8 ± 7.7 years). The prevalence of the risk factors considered (history of glaucoma or CV risk) was comparable in the 3 groups. Overall, the mean follow-up was 3.7 ± 2.1 years, with no statistically significant difference between the groups. BCVA loss in the nDTGA group was significantly less than in the other disease groups (*p* < 0.05). The clinical characteristics of the patients are summarized in Table 1.

**Table 1:** Clinical Characteristics of extensive macular atrophy with pseudodrusen-like appearance, diffuse-trickling geographic atrophy and geographic atrophy.

Categorical variables	EMAP (18)	DTGA (18)	nDTGA (27)	*p* value	
Number of females (%)	4 (22.2)	14 (77.8)	12 (44.4)	**0.02**	
CV risk factors (%)	8 (44.4)	5 (27.8)	18 (66.7)	0.35	
History of glaucoma (%)	0 (0)	0 (0)	2 (7.4)	0.33	

Quantitative variables				*p* value (DTGA vs EMAP)	*p* value (nDTGA vs EMAP)
Mean follow-up (years)	4 (1.6–6.0)	3.3 (2.5–4.9)	3.3 (2.0–5.0)		
Age at diagnosis (years)	55.4 ± 2.5	**65.4 ± 6.1**	**77.8 ± 7.7**	** < 0.01**	** < 0.01**
BCVA BL (logMAR)	0.5 ± 0.5	0.3 ± 0.4	0.5 ± 0.6		
BCVA FU (logMAR)	1.1 ± 0.5	0.8 ± 0.5	0.7 ± 0.6		
BCVA loss (logMAR)	0.6 ± 0.6	0.4 ± 0.5	**0.2 ± 0.4**	0.60	**0.02**

Categorical variables expressed as number (%). Normally distributed quantitative data expressed as mean ± standard deviation. Non-parametric quantitative data expressed as median (Interquartile range 25–Interquartile Range 75). *EMAP* Extensive macular atrophy with pseudodrusen-like appearance; *DTGA* Diffuse-trickling geographic atrophy; *nDTGA* non-diffuse-trickling geographic atrophy; *CV* Cardiovascular; *BCVA *Best Corrected Visual Acuity; *BL *Baseline; *FU *Follow-up.

*p* values of Chi-square test for categorical variables. *p* values of Tukey HSD post-hoc test for ANOVA for quantitative variable comparison with EMAP were reported if three-group comparison with ANOVA resulted statistically significant (*p* < 0.05).

Significant values are in bold.

### Area of atrophy

The area of atrophy at baseline and follow-up was significantly larger in EMAP than DTGA and nDTGA, but progression rates between the first two groups was similar: EMAP progression was faster than nDTGA (*p* < 0.05), but similar to DTGA. This was also true after the square root transformation of the progression rates. Regarding the shape of the lesions, baseline and follow-up CI was lower in EMAP than in nDTGA (*p* < 0.05), but not compared with DTGA (Table 2).

**Table 2:** Quantitative data of the atrophic area and choroidal parameters.

Atrophic area	EMAP (18)	DTGA (18)	nDTGA (27)	p EMAP vs DTGA	p EMAP versus nDTGA
Circularity index BL	0.1 ± 0.5	0.1 ± 0.6	0.3 ± 0.1*	**1.00**	** < 0.01**
Mean change	0.02 ± 0.06	0.06 ± 0.10	0.05 ± 0.2	0.77	0.70
Area of atrophy BL (mm^2^)	20.3 ± 12.7	8.7 ± 3.4*	6.0 ± 4.6*	** < 0.01**	** < 0.01**
Mean change (mm^2^)	1,7 ± 1.1	1.7 ± 0.9	0.9 ± 0.6*	1.00	**0.01**
Progression rate (mm^2^/year)	4.7 ± 2.4	3.9 ± 1.0	1.5 ± 0.7*	0.39	** < 0.01**
Squared progression rate (mm/year)	0.4 ± 0.2	0.5 ± 0.1	0.3 ± 0.1*	0.17	**0.02**
Choroidal parameters					
Subfoveal CT BL (µm)	165.5 ± 55.3	122.3 ± 35.7	141.4 ± 73.1	0.22	0.42
Mean change (µm)	− 34.9 ± 21.0	− 24.1 ± 17.2	− **18.5 ± 22.1**	0.43	**0.04**
Mean CT BL (µm)	156.3 ± 49.1	117.0 ± 32.6	117.3 ± 63.5	0.20	0.06
Mean change (µm)	− 20.4 ± 18.7	− 24.2 ± 16.5	− 13.6 ± 18.2	0.86	0.44
HLT BL (µm)	114.5 (98.0–135.0)	90.0 (83.0–96.0)	**63.0 (48.0–77.0) †**	0.20	** < 0.01**
Mean change (µm)	− 11.2 ± 18.8	− 19.1 ± 14.7	− 8.00 ± 15.8	0.48	0.80
SLCCT BL (µm)	38.0 ± 20.5	24.3 ± 16.6	43.7 ± 22.2	0.25	0.64
Mean change (µm)	− 9.2 ± 10.0	− 5.1 ± 4.2	− 5.3 ± 6.5	0.37	0.22
SCR BL	25.0 (17.9–30.0)	23.1 (14.0–25.2)	**38.1 (34.2 – 41.6) †**	0.46	** < 0.01**
Mean change	− 3.6 ± 5.7	− 0.1 ± 2.7	0.1 ± 6.4	0.30	0.10
CVI BL	0.7 (0.7–0.7)	0.7 (0.7–0.7)	0.7 (0.7–0.7)	0.38	0.79
Mean change	− 0.01 ± 0.05	− 0.01 ± 0.06	− 0.02 ± 0.06	1.00	0.71

Choroidal caverns	EMAP	DTGA	nDTGA	*p* value^aâ^	
Baseline	4 (22%)	2 (11%)	0 (0%)	** 0.04**	
Follow-up	8 (44%)	10 (56%)	0 (0%)	** < 0.01**	

Normally distributed data expressed as mean ± standard deviation. Non-parametric data expressed as Median (Interquartile range 25—Interquartile range 75). Qualitative data expressed as frequency (%). *EMAP* Extensive macular atrophy with pseudodrusen-like appearance; *DTGA *Diffuse-trickling geographic atrophy; *nDTGA* non-diffuse-trickling geographic atrophy; *BL* Baseline; *CT* Choroidal Thickness; *HLT* Haller layer thickness; *SLCCT* Sattler layer-choriocapillaris thickness; *SCR *Sattler/Choroid Ratio; *CVI *Choroidal Vascularity Index.

*p < .01 for the comparison with EMAP group using the Tukey HSD post-hoc test for ANOVA.

^†^*p* < .05 for the comparison with EMAP group using pairwise comparison for Kruskall-Wallis test.

^a^*p* value for Chi-square test.

Significant values are in bold.

### Choroidal parameters

Choroidal parameters at baseline were similar between the three groups, with the exception of baseline HLT and SCR: HLT was higher and SCR lower (both *p* < 0.01) in nDTGA than in the other two groups. Cavern frequency at baseline was lower in nDTGA than in EMAP and DTGA [4 (22%) in EMAP, 2 (11%) in DTGA, and none (0%) in nDTGA patients; *p* < 0.05). Interestingly, over the follow-up the frequency increased to 8 (44%) in EMAP, 10 (56%) in DTGA, and again zero (0%) in nDTGA (*p* < 0.001).

### Correlation analysis

Circularity at baseline also correlates with the progression rate before and after the square root transformation (all *p* < 0.001) and with SCR at baseline (r 0.553; *p* < 0.001). It is worth noting that subfoveal CT and CVI were the same in all three pathologies, while SCR was lower in EMAP and DTGA when compared with GA (*p* < 0.001). Also, CVI did not correlate with the rate of progression, while baseline SCR showed a negative correlation with both progression rate (r − 0.510; *p* < 0.001) and squared progression rate (r − 0.384, *p* < 0.001), as shown in Table 3. Finally, none of the considered parameters correlated with BCVA loss.

**Table 3:** Correlations between circularity, SCR and CVI and progression rates.

	Progression rate		Squared progression rate		BCVA loss	
	*R*	*p value*	*R*	*p value*	*R*	*p value*
SCR BL	− 0.51*	< 0.01	− 0.33*	**0.01**	− 0.05	0.43
CVI BL	0.10	0.52	0.12	0.38	− 0.01	0.62
Circularity BL	− **0.50†**	** < 0.01**	− **0.34†**	** < 0.01**	− 0.073	0.66

Correlation coefficients between the considered variables. *BCVA *Best corrected visual acuity; *SCR* Sattler/Choroid ratio; *BL *Baseline; *CVI* Choroidal Vascularity Index.

*p < .01 using Spearman’s correlation coefficient.

^†^ p < .05 using Pearson’s correlation coefficient.

Significant values are in bold.

Looking at the Bruch-RPE splitting, both EMAP and DTGA almost always display this feature, which is present in 18 cases (100%) of EMAP and 18 cases (100%) of DTGA at baseline and absent in 2 EMAP cases out of 18 at follow-up, on horizontal OCT scans. On the other hand, 10 (37%) cases of nDTGA showed this feature at baseline and 8 (30%) did so at the end of the follow-up (*p* < 0.001). When Bruch-RPE splitting disappeared over the course of the follow-up, RPE atrophy took its place.

Finally, EZ disruption distance was not evaluable in 4 (22%) EMAP patients, 6 (33%) DTGA patients and 2 (7%) nDTGA patients, and proved to be statistically different between the three groups at both baseline and follow-up (*p* < 0.001). EZ disruption distance data are reported in Table 4. It is worth noting that the disruption distance diminished over time in both EMAP and DTGA, while its progression was slightly positive in nDTGA phenotypes (Fig. 3). Intraclass correlation coefficient was 0.90 (0.79–0.95; *p* < 0.001).

**Table 4:** Ellipsoid Zone Disruption Distance from the atrophic area.

Group (n)	DD BL (µm)		DD FU (µm)		DD Progression (µm)	
	*Mean* ± *SD*	*p*	*Mean* ± *SD*	*p*	*Mean* ± *SD*	*p*
EMAP (18)	553 ± 407		408 ± 254		− 113 ± 474	
DTGA (18)	962 ± 510*	**0.01**	709 ± 397*	**0.02**	− 55 ± 307	0.92
nDTGA (10)	108 ± 66*	** < 0.01**	148 ± 119*	** < 0.01**	20 ± 88	0.42

Data expressed as mean ± standard deviation. *BL *Baseline; *FU *Follow-up; *DD* Disruption distance; *n *number of patients; *SD *standard deviation; *EMAP *Extensive macular atrophy with pseudodrusen-like appearance; *DTGA* Diffuse-trickling geographic atrophy; *nDTGA *non-diffuse-trickling geographic atrophy.

*p < .001 for the comparison with EMAP group using Tukey HSD post-hoc test for ANOVA.

Significant values are in bold.

## Discussion

Our study aimed to compare EMAP with GA subtypes, with an emphasis on the differences between the DTGA and the nDTGA phenotypes.

We found that atrophy enlarges faster in EMAP and DTGA than in nDTGA, even after the square root transformation of the progression rate, which is important since the atrophic area at baseline was larger in EMAP than in DTGA and nDTGA. We are not able to provide a definitive explanation for the higher baseline area in the EMAP group. However, in our experience, patients affected by EMAP tend to underestimate their initial symptoms (for example, nyctalopia) and usually require an ophthalmological examination very late in the course of the disease, when the atrophy gets closer to the macular region, leading to visual loss and paracentral or central scotoma.

Fleckenstein M. et al.^27^have already described similarities in the funduscopic appearance of DTGA and EMAP, hypothesizing a choroidal insufficiency as a potential pathogenetic mechanism, together with the presence of accumulated material between the RPE and Bruch’s membrane. Furthermore, also the choriocapillaris has also been implicated in dry AMD progression^28,29^. Recently, an association between CVI and GA growth has been reported^10^. Our research group also found that choroidal thinning takes place predominantly in the Sattler layer-choriocapillaris complex^11^.

In our cohort, CVI did not distinguish the three groups effectively, in contrast to the current literature^10^. However, other authors did not consider hyper- and hypo-transmission areas when assessing this parameter^30^. Our findings suggest it may be advisable to reach a consensus in order to calculate CVI.

By contrast, SCR is similar in EMAP and DTGA and higher in nDTGA, which might be an aid in diagnosing EMAP. The results of our investigation support the choroidal insufficiency theory and may indicate a primary involvement of SLCC. Additionally, SCR correlates with atrophy progression rates and circularity. The latter is another useful parameter in distinguishing EMAP and DTGA from nDTGA, and its correlation with the progression rate has already been described in the literature^5^. These findings suggest that the simultaneous presence of low circularity and SCR supports the diagnosis of EMAP or DTGA.

Finally, we detected choroidal caverns only in EMAP and DTGA; notably, their prevalence increased over the course of the follow-up. To this day, the histological and physiological nature of the phenomena behind these findings is a matter of debate. Some authors posit that choroidal caverns are “ghost vessels”^31,32^. However, on OCT scans choroidal caverns show a typical hypertransmission tail, an artifact known to be caused by lipid structures, such as the subretinal lipid globules described by Fernández-Avellaneda P. et al. in patients affected by neovascular AMD^33^. This finding is consistent with a recent study conducted by Dolz-Marco R. et al., who aimed to verify the correspondence of such choroidal caverns with the lipid globules first described by Friedman E. et al. in 1966^24,34^. Since they also found caverns in healthy patients, the authors hypothesized that they represent a normal aspect of the chorioretinal physiology, and that their persistence may reflect a slowdown in the photoreceptor metabolism ^24,34^. A more rapid decrease of the functional request may explain why we were able to identify choroidal caverns only in DTGA and EMAP patients. Moreover, the lipid globules previously mentioned are known to be more frequent in neovascular AMD, which was one of the exclusion criteria of the present study, than in GA due to AMD, and could explain their absence in our nDTGA group.

Both EMAP and DTGA display a diffuse involvement of the outer retinal layers, and the Bruch-RPE complex is split even in sites far from the atrophic regions. On the other hand, nDTGA does not always exhibit the Bruch-RPE splitting. Fleckenstein M. et al. have already described this feature in DTGA, and recently Romano F. et al. have also documented this OCT characteristic in EMAP^3,5^. We surmise that this phenomenon might be compatible with the presence of basal laminar deposits, which could in turn explain the grayish appearance of the atrophy on FAF images of EMAP and DTGA.

The presence of EZ disruption above the RPE splitting area may indicate that this event, which occurs after the accumulation of sub-RPE material, is of key importance in the progression of the disorder. The detection of EZ disruption in EMAP and DTGA even far away from the primary atrophic lesion is consistent with atrophy progression through separated foci. In these two groups disruption distance even decreases during the follow-up. Given that we took this measurement on horizontal scans, this result is consonant with the prevalent vertical progression of the atrophy, although we could not evaluate vertical scans because of the limited field of view. By contrast, in nDTGA the EZ always fades close to the borders of the atrophy, supporting the hypothesis that Bruch-RPE splitting is a key event in the pathogenesis of both EMAP and DTGA.

We are aware that our investigation is burdened by several limitations. Firstly, our sample was relatively small, owing to the problems related to the patient referral in the absence of any available therapy, and due to the rarity of EMAP, which could explain both the unusual distribution of males and females in our cohort and the absence of choroidal caverns in the nDTGA group. Moreover, in almost 20% of cases the follow-up was uneven, due to COVID-19 restrictions. In addition, although unlikely, rare forms of macular atrophy (such as Sorsby fundus dystrophy) could not be excluded, since we did not carry specific genetic testing on our sample.

In our analyses, we excluded areas of hyper- and hypo-transmission in our measurements, although CVI is not affected by the region considered^15^. Besides, CVI is yet to be standardized, which impacts on the reproducibility of our data. In addition, EZ disruption distance is a novel parameter, intended as a proxy of the extension of the Bruch-RPE splitting, which has several shortcomings. The EZ was occasionally not clearly evaluable or not visible at all due to the technical limitations of the OCT resolution and field of view. We therefore arbitrarily chose to measure the EZ disruption distance on the temporal side of the horizontal scan, in an attempt to ensure a valid reproducibility.

In conclusion, EMAP and DTGA are known to be the most aggressive forms of macular atrophy in terms of expansion and visual acuity deterioration and share several features, suggesting a common pathogenetic background. It is unclear whether DTGA should be regarded as a late-onset EMAP-like disease. When it comes to developing concrete, feasible treatments in the near future, it might be helpful to distinguish more clearly the different forms of macular atrophy, so as to optimize dry AMD and EMAP patient management. Further studies with larger samples and longitudinal follow-ups are warranted to expand our knowledge of the different macular atrophy phenotypes.

## Financial disclosures

Francesco Bandello is consultant for Alcon (Fort Worth, Texas, USA), Alimera Sciences (Alpharetta, Georgia, USA), Allergan Inc (Irvine, California,USA), Farmila-Thea (Clermont-Ferrand, France), Bausch & Lomb (Rochester, New York, USA), Genentech (San Francisco, California, USA), Hoffmann-La-Roche (Basel, Switzerland), Novagali Pharma (Évry, France), Novartis (Basel, Switzerland), Bayer Shering-Pharma (Berlin, Germany), Sanofi-Aventis (Paris, France), Thrombogenics (Heverlee, Belgium), Zeiss (Dublin, USA), Pfizer (New York, USA), Sanofi-Aventis (Paris, France), Santen (Osaka, Japan), Sifi (Aci Sant’Antonio, Italy), Thrombogenics (Heverlee, Belgium), Zeiss (Dublin, USA). All other authors have no financial disclosure.

## Data Availability

The datasets used and/or analysed during the current study available from the corresponding author on reasonable request.
